# Supplementary material for: Identification and Characterization of the BBX Gene Family in Bambusa pervariabilis × Dendrocalamopsis grandis and Their Potential Role under Adverse Environmental Stresses
Source: Int J Mol Sci. 2023 Aug 30;24(17):13465. doi: 10.3390/ijms241713465 (PMC10488121; doi:10.3390/ijms241713465)
Supplement: Supplementary file 1 [file ijms-24-13465-s001.zip › ijms-2530364-supplementary.pdf]

Supplementary Materials

Table S1. qPCR primers for 21 *BDBBX* genes.

Primer Name	Primer Sequence 5'-3'	
BDBBX1-F	CGACATCTGCCAGGTGAGAG	
BDBBX1-R	AGGAACCCCCTCTTCTCCTG	
BDBBX2-F	CAGAACGCCCAGGACTTCTT	
BDBBX2-R	GGTTCTCCGGGGAGCATATG	
BDBBX3-F	TTGGGTTCAAGGAGCTGGAC	GGTTCAAGGAGCTGGACTGG
BDBBX3-R	ACCCGTGACTTCTTGCTCTG	ACCGCTCGTCTTGTAGAAGC
BDBBX4-F	TAACCACAAACGGCATTGGC	
BDBBX4-R	TGCTGTTCCCGTCAACAAGT	
BDBBX5-F	TCGTCACCTCCTGAAACACG	
BDBBX5-R	ACGCAGACTCGCTCGTGATC	
BDBBX6-F	AACGTGGATGCCAAGGACAT	
BDBBX6-R	CGTGTAGGAGCTGTAGGACG	
BDBBX7-F	TGCACTCCGCCAACAAGCT	TTGCACGTGTGGGGTTCATA
BDBBX7-R	GTACCTGGCAGATGTCGCAG	GTCAGGGTAGTTTGGTGGCA
BDBBX8-F	CTTTCCTGCGATGCCAAGGT	GACAAGGCTCCGGAGTTTGA
BDBBX8-R	CGGTCGACATGCAAGAACTG	AGGAAAGGCAGAGGAACGC
BDBBX9-F	TCCGAGGAGGAGAACAGCAG	GCAGCATCTCCGAGTACCTG
BDBBX9-R	CATCGTCGACCAGGAAGTCC	GTCTGCAGAGATGCCGGTAG
BDBBX10-F	GCCAAAGTGCGACATATGCC	
BDBBX10-R	GGTCCTCGAGGCAGAAGAAG	
BDBBX11-F	AGGAGTCTCCTCTCGGGTTC	
BDBBX11-R	CTTGTAGAAACCCGCGTTGC	
BDBBX12-F	CAGAACGCCCAGGACTTCTT	
BDBBX12-R	GGTTCTCCGGGGAGCATATG	
BDBBX13-F	AGTTCCTCTGCTCCAACTGC	
BDBBX13-R	CTTCTCGAACTCGGGGACAC	
BDBBX14-F	CTACGAGTCCAGCGACAAGG	
BDBBX14-R	CAGCTCCTTGAACCCGAGAG	
BDBBX15-F	ATAGAGGAGGCGGAGGAGGA	
BDBBX15-R	CGTCTTGGTCAGGTACTCGG	
BDBBX16-F	AGCTATACAATCCCGTGCCC	AGCTATACAATCCCGTGCCC
BDBBX16-R	CTAGCAAGCTCCATGTCCGG	CTAGCAAGCTCCATGTCCGG
BDBBX17-F	CTCTTCTGCTCCGACCACTC	
BDBBX17-R	GCTTCTCTCGGTACCGCATC	
BDBBX18-F	CCTGGGGTTCTTGGACGAAG	
BDBBX18-R	ATCTCCGGCCGCTTCTTTTT	
BDBBX19-F	TGGAGCTGCACAAGTACTGG	
BDBBX19-R	CAGACCTCGCACATCCACAC	
BDBBX20-F	TCGTACCCACCACTTCTTGC	TCGTACCCACCACTTCTTGC

BDBBX20-R	GTAGCGTGAAATCACCGTGC	GTAGCGTGAAATCACCGTGC
BDBBX21-F	CTCCACTTGGGTTCACGGAG	
BDBBX21-R	TCGTCTTGTAGAAACCGGCG	

Table S2. Disease grading criteria.

Degree	Grading criteria
0 level	no disease observed on the leaf
1 level	disease spots accounting for <30% of the total area
2 level	disease spots accounting for 30 to 50% of the total area
3 level	disease spots accounting for >50% of the total area
4 level	withered leaves

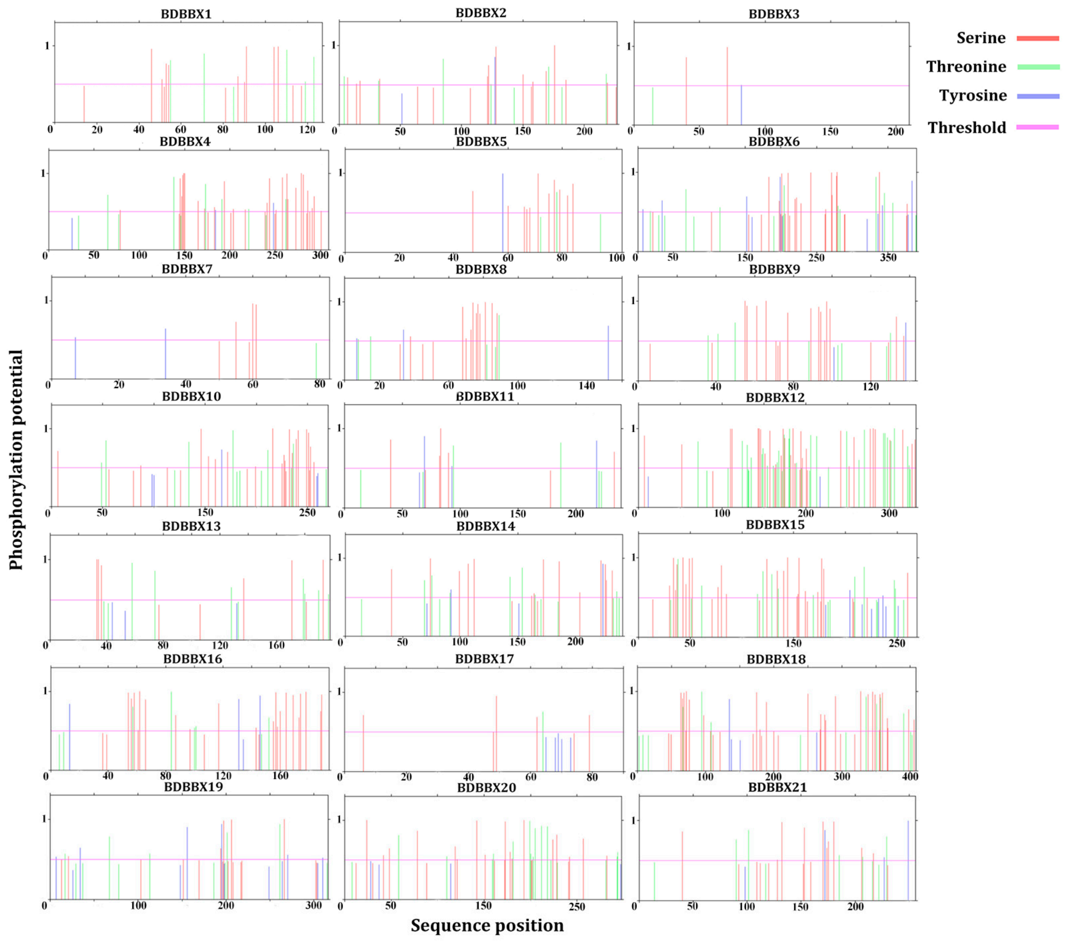

Figure S1. Potential phosphorylation sites for the *BDBBX* gene family.

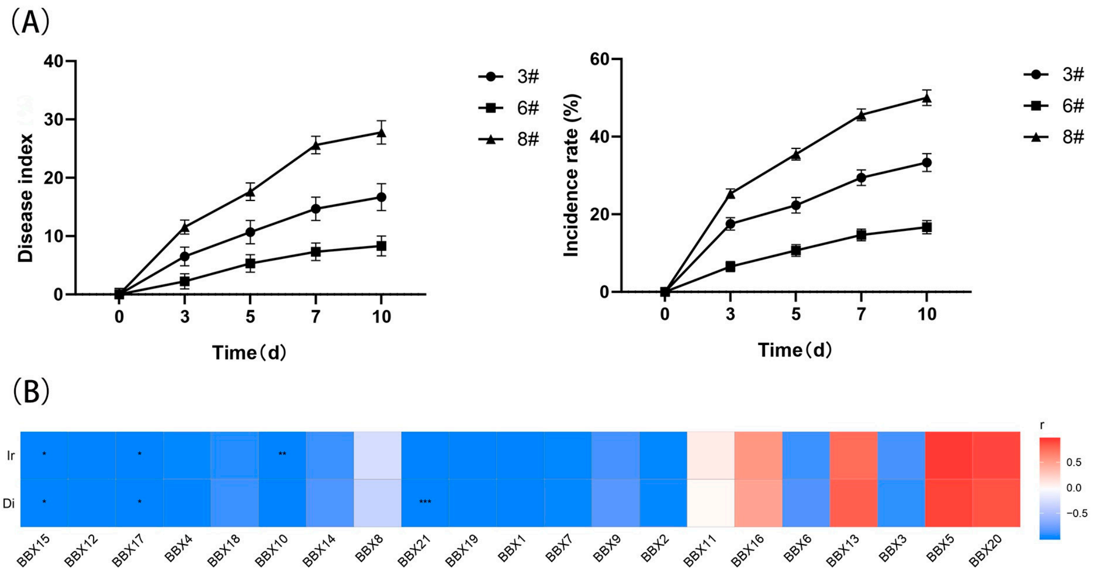

Figure S2. (**A**) Disease index of three *B. pervariabilis* × *D. grandis* varieties; (**B**) Correlation analysis of gene expression with incidence rate (Ir) and disease index (Di). * indicates p value less than 0.05, ** indicates p value less than 0.01, and *** indicates p value less than 0.001.
